# *In situ* genotyping of a pooled strain library after characterizing complex phenotypes

**DOI:** 10.15252/msb.20177951

**Published:** 2017-10-17

**Authors:** Michael J Lawson, Daniel Camsund, Jimmy Larsson, Özden Baltekin, David Fange, Johan Elf

**Affiliations:** ^1^ Department of Cell and Molecular Biology Science for Life Laboratory Uppsala University Uppsala Sweden

**Keywords:** DuMPLING, live cell, microfluidic, single cell, strain libraries, Methods & Resources, Quantitative Biology & Dynamical Systems

## Abstract

In this work, we present a proof‐of‐principle experiment that extends advanced live cell microscopy to the scale of pool‐generated strain libraries. We achieve this by identifying the genotypes for individual cells *in situ* after a detailed characterization of the phenotype. The principle is demonstrated by single‐molecule fluorescence time‐lapse imaging of *Escherichia coli* strains harboring barcoded plasmids that express a sgRNA which suppresses different genes in the *E. coli* genome through dCas9 interference. In general, the method solves the problem of characterizing complex dynamic phenotypes for diverse genetic libraries of cell strains. For example, it allows screens of how changes in regulatory or coding sequences impact the temporal expression, location, or function of a gene product, or how the altered expression of a set of genes impacts the intracellular dynamics of a labeled reporter.

## Introduction

Recent years have seen a rapid development in genome engineering, which, in combination with decreased costs for DNA oligonucleotide synthesis, have made it possible to design and produce pool‐generated cell libraries with overwhelming genetic diversity (Wang *et al*, 2009; Dixit *et al*, 2016; Jaitin *et al*, 2016; Peters *et al*, 2016; Garst *et al*, 2017; Otoupal *et al*, 2017). A similarly impressive development in microscopy enables the investigation of complex phenotypes at high temporal resolution and spatial precision in living cells (Liu *et al*, 2015; Balzarotti *et al*, 2017). Biological imaging has benefited greatly from developments in microfluidics which have enabled well‐controlled single‐cell observations of individual strains over many generations (Wang *et al*, 2010; Uphoff *et al*, 2016; Wallden *et al*, 2016). Despite the rapid technological progress within these areas, there is currently no efficient technique for mapping phenotypes related to intracellular dynamics or localization to their corresponding genotype for pool‐generated libraries of genetically different cell strains. Recent work observing multiple bacterial strains on agarose pads allows for sensitive microscopy (Kuwada *et al*, 2015; Shi *et al*, 2017), but the genetic diversity is capped since the strain production and handling is not pooled. On the other end, droplet fluidics allows working with large genetic diversity (Dixit *et al*, 2016) but cannot be used to characterize phenotypes that require sensitive time‐lapse imaging.

Here, we present a method that solves the problem by *in situ* genotyping the library of strains after the phenotypes have been studied in time‐lapse microscopy. Thus, the genotype of the cell is not known at the time of phenotyping but revealed through the spatial position of the cell after fixation and *in situ* genotyping.

## Results

### The DuMPLING approach

**Figure 1:**
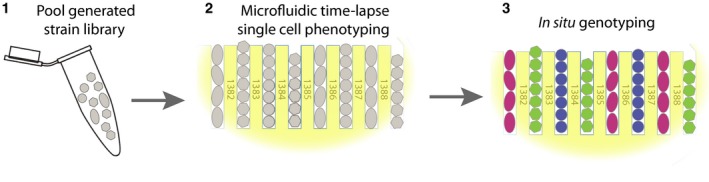
The DuMPLING strategy (1) Pooled strain library generation. (2) Live single cell phenotyping using microscopy. (3) Genotypes recovered by *in situ* genotyping.

**Figure 2:**
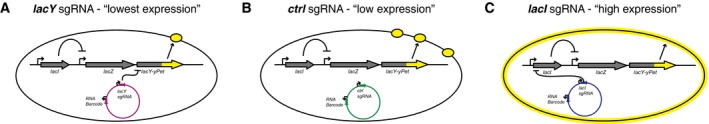
Three strain *lac* operon knockdown library: Repression network for the three different plasmids used *lacY* knockdown (lowest LacY‐YPet expression, purple).No knockdown (low LacY‐YPet expression, green).
*lacI* knockdown (high LacY‐YPet expression, blue).Data information: Color scheme holds throughout this paper.

**Figure EV1:**
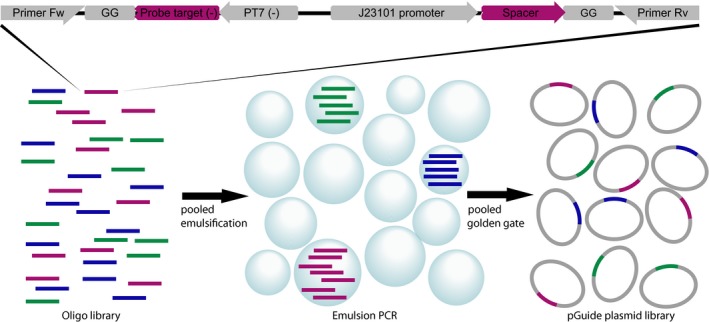
DuMPLING oligonucleotide plasmid library design and production Each member of the oligonucleotide library for plasmid construction contains two divergent promoters: a constitutive promoter toward the spacer and PT7 toward the barcode RNA (top). The oligonucleotide library was amplified using pooled emulsion PCR to avoid the formation of chimeras (bottom middle). The pool of amplified oligonucleotides was assembled into a functional pGuide plasmid library using pooled Golden Gate (bottom right).

**Figure EV2:**
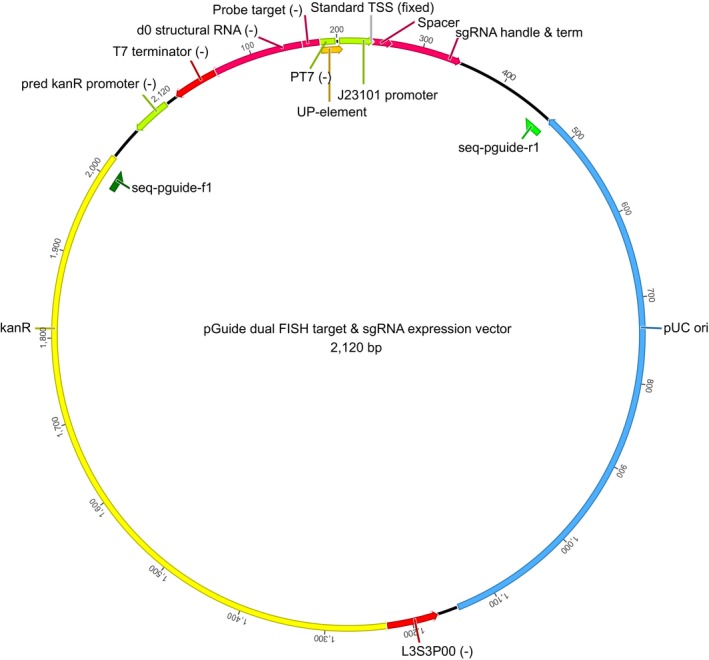
Sequence elements of the dual RNA barcode FISH target and sgRNA expression pGuide vector Minus (−) signs indicate anti‐sense direction of genetic element.

**Figure 3:**
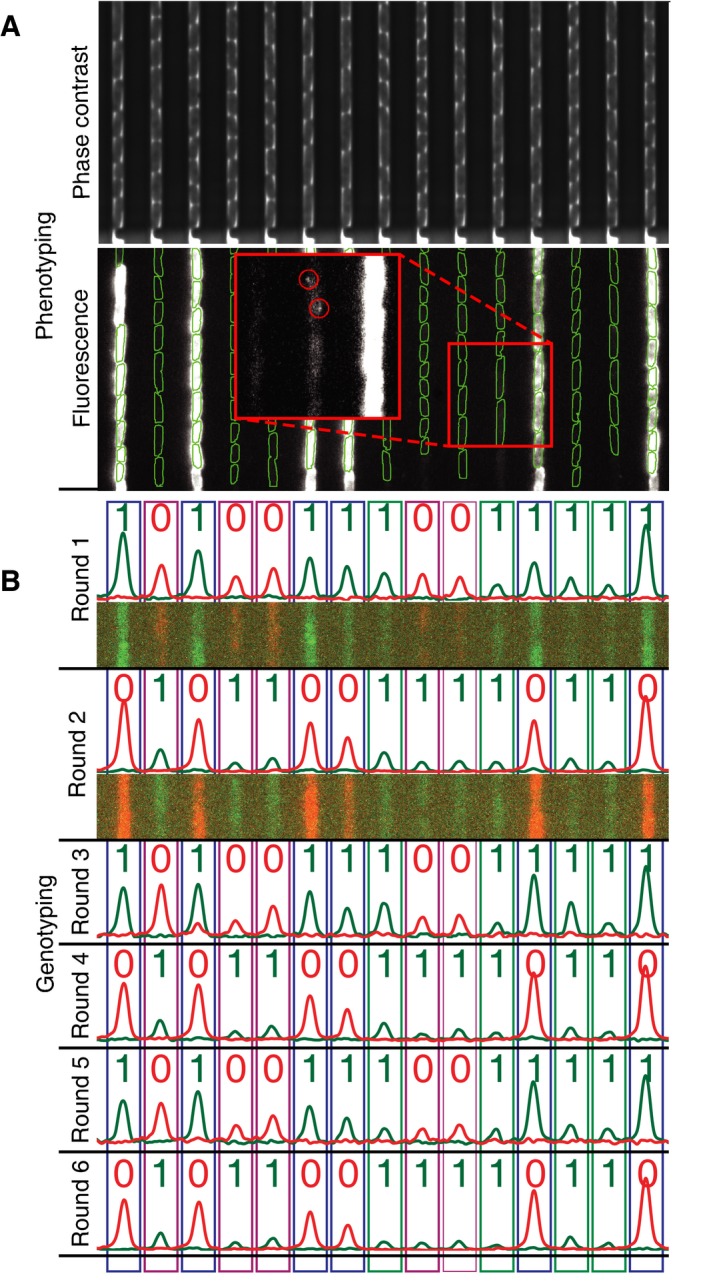
Mapping phenotypes to genotypes Examples of channels and cells in the custom‐made microfluidic device which are imaged in both phase contrast (top) and fluorescence microscopy (bottom). Phase contrast is used to segment the cells (green outlines), and single‐molecule fluorescence microscopy is used to detect gene expression (red circles in red inset box, which is a blow up of the figure as indicated by the smaller red square and has a change of levels to allow visualization of single molecules) from the *lac* operon.
*In situ* genotyping with six sequential rounds of FISH probe hybridization and stripping. Cropped images of two cells that are representative of all cells in the trap are shown for the first two rounds (overlay of Cy3 (green) and Cy5 (red) images). The genotype is called by summing the signal in the channel: 0 is assigned for Cy5 (red) and 1 for Cy3 (green). Rectangles indicate assigned genotype (10: lacI knockdown; 01: lacY knockdown; 11: no knockdown).

We refer to our solution of the library phenotyping problem as DuMPLING—**d**ynamic **u**‐fluidic **m**icroscopy‐based **p**henotyping of a **l**ibrary before ***in** situ* **g**enotyping. DuMPLING is composed of three key components: strain generation, live cell phenotyping, and *in situ* genotyping (schematically outlined in Fig 1). All three components can be made in different ways, but in the current study, we have selected this implementation:

1. *Pool‐generated strain library:* We have constructed a library of CRISPRi/dCas9 knockdowns. We generated a recipient strain harboring chromosomal inducibly expressed dCas9 and T7 polymerase. We used Golden Gate assembly to generate a small plasmid‐expressed library of sgRNA spacers (to direct the dCas9 chromosomal binding and create knockdowns) and neighboring barcode sequences (for later genetic identification) (Figs 2, EV1, and EV2). Note that in 167 nt, we fit the variable regions (i.e., the barcode sequence and sgRNA spacer sequence), the constant elements between the variable regions and the constant regions on the ends for PCR and assembly (see Supplement for sequence design details). This length of oligo is easily procured from companies, and much larger libraries have been built following this approach with purchased oligo pools (Dixit *et al*, 2016), making it clear that this strategy of library construction can be extended to a genomewide knockdown library.
2. *Live cell phenotyping in a microfluidic device where each strain occupies a defined position:* The mixed strains are loaded into a microfluidic chip which harbors 4,000 cell channels, sustains continuous exponential growth, and allows single‐cell imaging for days (Fig 3A, Movies EV1 and EV2). After only a few generations, all cells in a channel are the progeny of the cell at the back of the channel and thus share the same genotype. The chip design is similar to the mother machine (Wang *et al*, 2010), but we have introduced a 300 nm opening in the back of each cell channel such that media and reagents can be passed over the cells. This redesign facilitates cell loading and is essential for genotyping.
3. *In situ genotyping to identify which strain is in which position*: As mentioned above, each plasmid expresses a unique RNA‐based barcode that allows genotype identification. The barcode is expressed from a T7 promoter, and the T7 polymerase is under control of an inducible arabinose promoter. The orthogonal and inducible nature of this system prevents it from interfering with cell physiology during phenotyping. After induction of the barcode RNA expression, the cells are fixed *in situ* with formaldehyde and permeabilized in 70% EtOH before sequential fluorescent *in situ* hybridization (FISH). The individual barcodes are identified by sequential hybridization of fluorescent 37‐nt‐long oligonucleotides (probes). The multiplexed process of designing and producing the probe library is described in the Materials and Methods section. The templates for probe synthesis are procured in the same array format as the barcoded sgRNA templates. Here, we use probes of two different colors in two sequential rounds of probing, which is sufficient for identifying the three genotypes in this study.

In general, C^*N*^ genotypes can be identified where C is the number of colors and *N* is the number of rounds of probing. Thus, genotyping can straightforwardly be extended to more strains by using more colors or rounds of probing. For example, a recent publication (Shah *et al*, 2016) showed four rounds of single‐molecule FISH probing in five colors (i.e., 625 genotypes), and they observed a miss‐call rate of ~1%. We would however expect a lower error rate than this as we are imaging ~6 cells of the same genotype, each containing many RNA rather than individual RNA molecules. To demonstrate that it is possible to reprobe many times, we perform *N* = 6 consecutive rounds (Fig 3B) of probing in each position. It is however likely that more rounds are possible without loss of specificity. For example, in a recent study, Chen *et al* were able to successfully probe single RNA molecules 16 rounds (Chen *et al*, 2015).

### Proof‐of‐concept demonstration

**Figure EV3:**
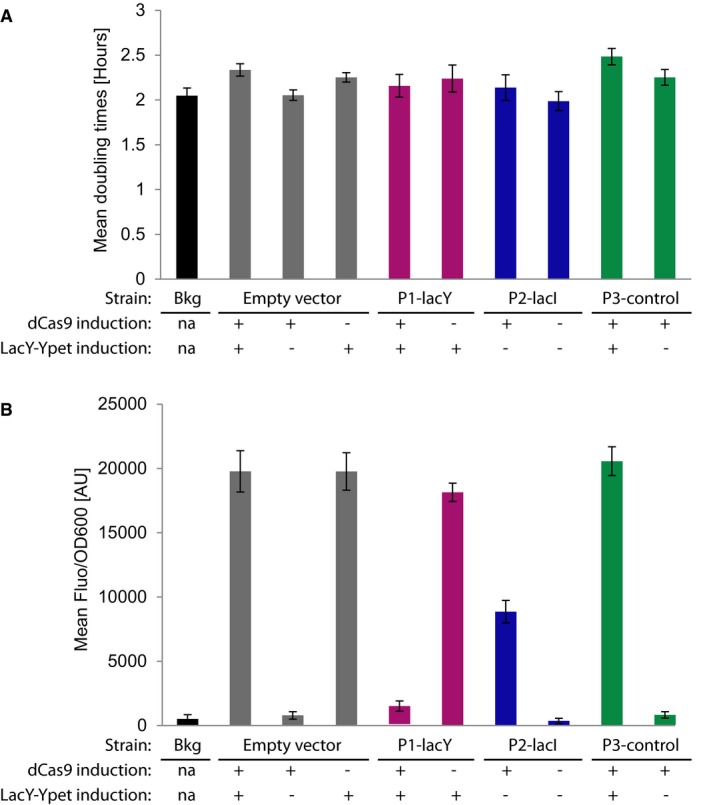
Bulk growth rate and CRISPRi repression assay results of the DuMPLING screening strain with different pGuide constructs Mean doubling times of the different strains under the indicated conditions.Steady‐state mean fluorescence normalized by cell density (Fluo/OD_600_) of the different strains under the indicated conditions.Data information: Error bars indicate sample standard deviations (Bkg *n* = 3, Empty vector *n* = 3, P1‐lacY and P2‐lacI *n* = 6, P3‐control *n* = 5). Bkg (cell background), AU (arbitrary units), na (not applicable).

To exemplify the use of DuMPLING, we performed targeted knockdowns of different components of the *lac* operon in *Escherichia coli* using a set of sgRNA‐expressing plasmids that repressed *lacY*, an unrelated gene or *lacI* (Fig 2A–C). As described above, the plasmids are made from pooled oligos including the sgRNA and its unique barcode. The pooled approach has previously been used to generate libraries of thousands of genotypes (Dixit *et al*, 2016; Jaitin *et al*, 2016), but here, we limit to three variants to be able to precisely evaluate the accuracy of each step. The mixed plasmids are electroporated into an *E. coli* strain, where dCas9 is expressed from a regulated chromosomal promoter (the promoter is tightly regulated to prevent bias in growth before loading and induction, Fig EV3). Furthermore, the *lacY* gene is fused with the gene for the fluorescent protein YPet to obtain a detectable single‐molecule phenotype.

In our experiments, 233 channels are imaged every 60 s using phase contrast and every 13 min using single‐molecule‐sensitive wide‐field fluorescence for a total of 272 min. Phase contrast images are used for cell detection and lineage tracking. Individual LacY‐YPet molecules, detected using wide‐field epifluorescence, are overlaid on the phase contrast images to allow assignment of individual molecules to individual cells.

**Figure 4:**
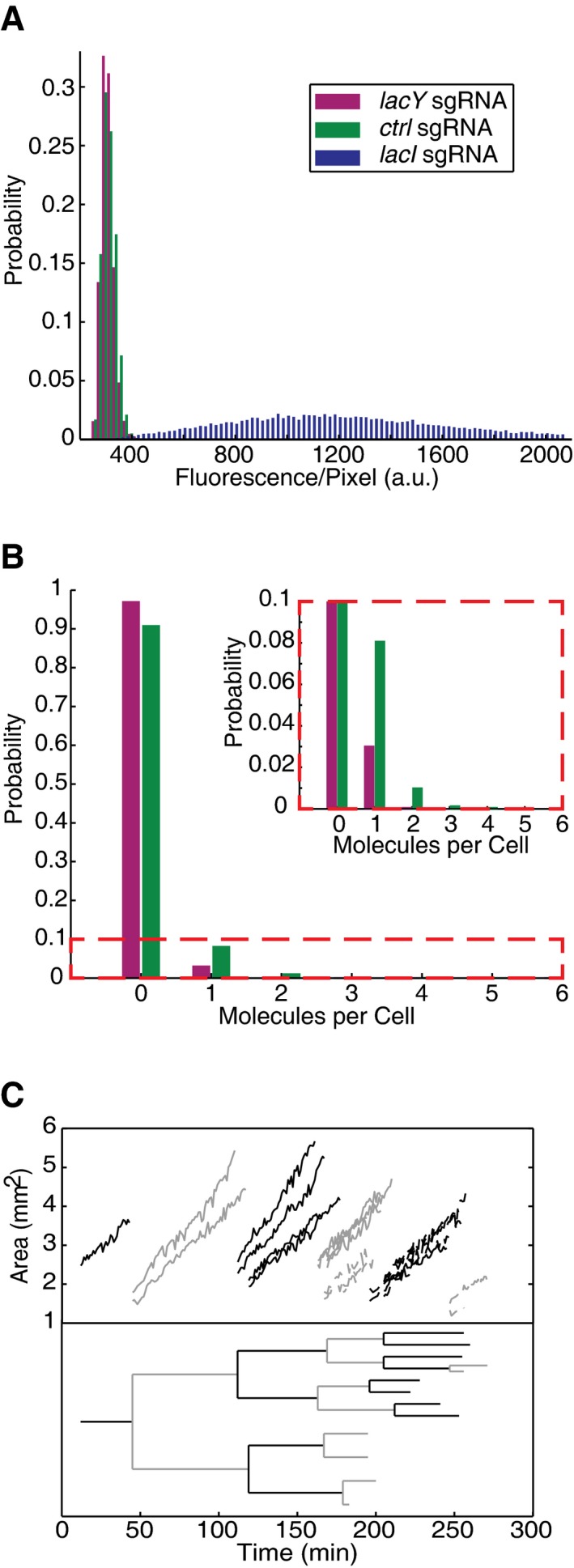
Phenotype data Gene expression categorized by assigned genotype.Single‐molecule counting of expression from the two low‐expression genotypes.Top growth curves for one cell lineage (from one channel). Dashed lines indicate the end of detection of a branch. Bottom corresponding lineage tree.

**Figure EV4:**
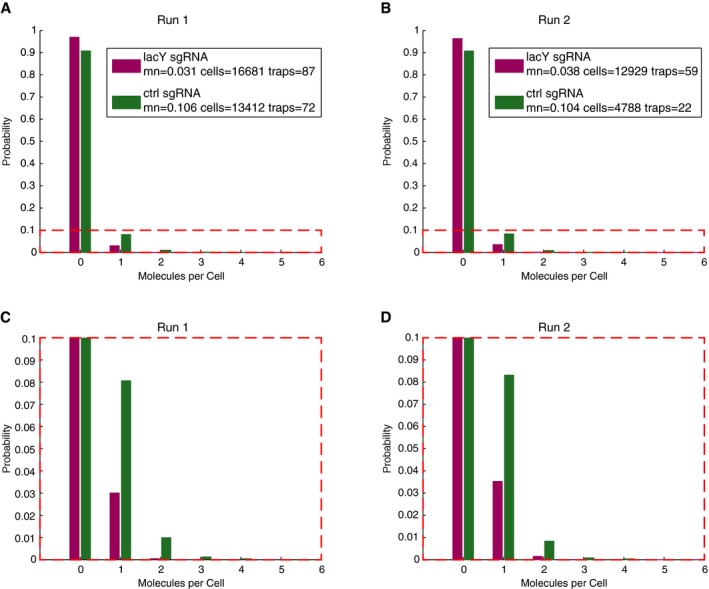
Reproducibility of dot detection results A–DNormalized histograms of single‐molecule counting of expression from the two low‐expression genotypes (strain definitions are given in the Materials and Methods section “Design and construction of the CRISPRi/RNA barcode plasmid library”). (A and C) are the same results displayed in Fig 4B. (B and D) are from a repeat of the same experiment 1 week later. Note: (A and B) are the full histogram, (C and D) are zoom in on the lower frequency events. mn: mean.

We were able to track a cell lineage over the full time‐course of the experiment (six generations) and quantify the growth curves of each member of the family tree (see example in Fig 4C). In addition, the long time course of single‐cell/single‐molecule microscopy allowed us to reproducibly measure mean expression of less than one YPet molecule per generation and distinguish a < 3× change at this expression level (compare distribution of single‐molecule counts per cell in Figs 4B and EV4). This type of phenotyping is not possible in most other settings (e.g., flow cytometry) and would not scale to hundreds of strains in those where it is possible (e.g., agarose pads mounted on a microscope).

While the phenotypic difference between the two low‐expression strains can only be resolved with extensive single‐molecule time‐lapse imaging, we also included the *lacI* knockdown phenotype, which is trivial to identify, to test for correct genotype to phenotype assignments. All 74 channels with cells that express a high level of LacY‐YPet (Fig 3A) have been correctly found to express the barcode RNA associated with the sgRNA against *lacI* (blue boxes in Fig 3B and blue bars in Fig 4A), and all channels with cells with the barcode RNA associated with the sgRNA against *lacI* express high levels of LacY‐YPet. The observed sensitivity and specificity for identifying the genotype in this experiment is therefore 100%. If we also consider the limited sample size and the redundant genotyping as independent, the sensitivity is > 97.5% and the specificity > 99% (see Materials and Methods section for details).

## Discussion

This paper describes a proof‐of‐principle application of the DuMPLING concept, that is, the possibility to use advanced microscopy to phenotype a pool‐generated library of live cells and then genotype *in situ*. The advantage of our method compared to the state of the art is the combination of pooled handling of library generation and characterization of complex phenotypes based on dynamic changes in single cells. We have used a microfluidic device to both phenotype the cells in a constant growth environment for an extended period of time and perform the subsequent genotyping.

We note that each of the components (strain library generation, phenotyping, and genotyping) can be performed in different ways depending on the specific question. For example, one can make pooled dCas9 libraries based on plasmids harboring both a genotype‐identifying barcode and a sgRNA gene (Dixit *et al*, 2016; Jaitin *et al*, 2016; Peters *et al*, 2016; Garst *et al*, 2017; Otoupal *et al*, 2017) for labeling genetic loci (Chen *et al*, 2013) or knocking down/activating genes throughout the chromosome. Alternatively, pooled chromosomal libraries with variants of promoters, ribosome binding sites (RBS), or coding sequences (Wang *et al*, 2009; Keren *et al*, 2016) can be made. Furthermore, it is in general not necessary to introduce the barcode in direct proximity to the genetic alterations as long as the barcode can be connected to the genotype in some other way than through the oligo synthesis. For example, long sequence reads can connect random barcodes to the genetic alteration that causes a phenotype.

Similarly, sensitive single‐cell time‐lapse imaging can be used to characterize a bewildering diversity of cell phenotypes than are not accessible with snapshot measurement as obtained in FACS or droplet fluidics (Norman *et al*, 2013; Hammar *et al*, 2014; Taheri‐Araghi *et al*, 2015; Potvin‐Trottier *et al*, 2016; Wallden *et al*, 2016). Depending on the cell types and the experiment, it may also be more convenient to use an open culture dish instead of the fluidic device.

Also, the method for identifying the barcode can be implemented in different ways such as *in situ* sequencing (Ke *et al*, 2013; Lee *et al*, 2014). One advantage of direct *in situ* sequencing is that the genotype may be identified directly without the use of a barcode.

In short, while we have presented a CRISP‐FISH‐DuMPLING, the DuMPLING can have many other fillings.

## Materials and Methods

### Design and construction of the DuMPLING screening strain

The dCas9 expression cassette from Qi *et al* (2013), which includes the TetR repressor and the bidirectional PRPA promoter that regulates both *tetR* and *dcas9*, was introduced into the chromosome of *E. coli* and optimized for low leakage of dCas9 under non‐induced conditions.

Briefly, the dCas9 expression cassette (plasmid pdcas9 Addgene 44249) and a spectinomycin resistance (SpecR) cassette were separately amplified using Phusion polymerase (Thermo Scientific, all PCRs were performed with Phusion unless otherwise specified) (primers: revL3S2P11‐tetR‐f1, olp‐rrnBTwP(SpR)‐r1; olp‐rrnBTwP(SpR)‐f1, L3S2P55‐SpR‐r1) and fused together via overlap PCR. Fragments from *intC* were separately amplified and fused together via overlap PCR to enable chromosomal recombination (primers revIntC‐f1, olp‐smaI‐revIntC‐r1; olp‐smaI‐revIntC‐f2, revIntC‐r2). The fusion was digested with SmaI, and the dCas9‐SpecR cassette was inserted [cloning steps were performed in the pGEM‐T easy (Promega) vector and confirmed with Sanger sequencing (Eurofins Genomics)].

The construct was subcloned into the NotI site of pKO3 (Link *et al*, 1997), inserted into *intC* of *E. coli* BW25993 using double recombination as previously described (Link *et al*, 1997), and sequence‐verified. Finally, the complete, integrated construct was transferred by P1 phage generalized transduction to a BW25993 strain carrying a translational fusion of *ypet* to *lacY* in the native *lacZYA* operon, generating strain BW25993 *intC::tetR‐dcas9‐aadA lacY::ypet‐cat*.

#### The TetR and dCas9 promoters were optimized to minimize non‐induction leakage

First, the PRPA bidirectional promoter, driving the expression of both TetR and dCas9, was replaced with two separate promoters driving each gene. The PLtetO‐1 promoter (Lutz & Bujard, 1997) and a strong synthetic RBS designed using the RBS calculator (Salis *et al*, 2009; Espah Borujeni *et al*, 2014) were used for regulating the expression of dCas9. For driving the expression of TetR, the combined promoter and RBS sequence element PN25 was used (Lutz & Bujard, 1997). However, the dCas9 leakage levels were not sufficiently low, so PN25 was switched out for the stronger proB TetR expression elements (Rogers *et al*, 2015) in a second step. The dCas9 and TetR promoter engineering was made in the BW25993 *intC::tetR‐dcas9‐aadA lacY::ypet‐cat* strain with λ‐RED recombination using the pKD46 plasmid (Datsenko & Wanner, 2000). The PRPA promoter region was exchanged with a kanamycin resistance (kanR)‐sacB cassette produced using Phusion polymerase and primers URStetR‐kanRsacR‐f1 and DRSdcas9‐kanRsacR‐r1. For producing the PN25‐PLtetO‐1 recombination fragment, oligonucleotides PN25‐SpOLP and SpOLP‐PLtet‐strRBS were fused using PrimeSTAR polymerase (Takara) overlap extension, and the product was used in a PrimeSTAR PCR with primers URStetR‐PN25‐f1 and DRSdcas9‐strRBS‐r1. The integrated kanR‐sacB cassette was exchanged with the PN25‐PLtetO‐1 recombination fragment. To engineer stronger TetR expression, the PN25 expression region was first exchanged with a kanR‐sacB cassette amplified using primers URStetR‐kanRsacR‐f1 and DRS‐spacer‐kanRsacR‐r1. The proB recombination fragment was generated by fusing the two oligonucleotides tetR‐Rogers‐spac‐oligo1 and tetR‐Rogers‐spac‐oligo2 using PrimeSTAR. Finally, the integrated kanR‐sacB cassette was exchanged with the proB recombination fragment to produce the final dCas9 construct, and all modified regions were confirmed by sequencing. The completed dCas9 construct was transduced to a fresh *lacY::ypet‐cat* strain.

To complete the DuMPLING screening strain, the T7 RNA polymerase gene regulated by the *araBAD* promoter was P1 phage transduced from the BL21 AI strain (Invitrogen), producing the final BW25993 *intC::tetR‐dcas9‐aadA lacY::ypet‐cat araB::T7 RNAP‐tetA* Δ*araB* strain with the new name EL101. All oligonucleotides used for cloning are available in Table EV1.

### Design and construction of the CRISPRi/RNA barcode plasmid library

To directly connect each unique sgRNA spacer with a specific barcode, the sgRNA and the barcode were placed in close proximity and expressed from divergent promoters (Fig EV1). This makes it possible to fit both the variable part of the sgRNA and the barcode within the current commercial synthesis limit. The sgRNA and the barcode RNA are driven by a constitutive sigma70 promoter and a T7 promoter, respectively. The two expression units are separated by a spacer that positions the UP‐element of the sgRNA promoter in the constant region of the RNA barcode promoter. The final construct, including flanking priming regions to facilitate cloning, came to 167 nt.

Each of the three library oligonucleotides (probe1‐lacY‐spacer (P1‐lacY), probe2‐lacI‐spacer (P2‐lacI), and probe3‐control‐spacer (P3‐control)) consists of a unique FISH RNA barcode sequence paired with a unique sgRNA spacer targeting *lacY*,* lacI,* and a control spacer with five mismatches toward *ypet*, respectively. To avoid the formation of chimeras, emulsion PCR, with library oligos sufficiently diluted to ensure that each droplet at most contains one template, was used (Williams *et al*, 2006; Shao *et al*, 2011; Fig EV1). The PCR was performed with DreamTaq on templates in a 1:5 ratio with the expected number of emulsion droplets (primers library‐fw and library‐rv). The emulsion PCR product was recovered (Shao *et al*, 2011) and then purified using a commercial kit (Purelink Quick PCR Purification Kit, Invitrogen). The pGuide backbone was PCR‐amplified to introduce GG adapters with Q5 DNA polymerase (primers forward pGuide3‐early and BpiI‐pG7‐d0‐rv). The PCR product was DpnI‐treated and gel‐purified (Purelink Quick Gel Extraction Kit, Invitrogen). The Golden Gate assembly was carried out using BpiI and an approximately 1:1 molecular ratio of amplified pooled library DNA and pGuide backbone product (Engler & Marillonnet, 2013). The assembled pGuide plasmid library was purified (PCR Purification Kit, Invitrogen) and electroporated into the DuMPLING screening strain. After recovery, the cell library was either selected in liquid media (LB + 50 μg/ml kanamycin at 37°C for 3 h) to make cryostocks or plated on LB agar + 50 μg/ml kanamycin plates for estimating library construction accuracy and diversity.

Library colony PCR was carried out using DreamTaq polymerase and the seq‐pguide‐f1 and r1 primers. Sequence verification of 24 colonies confirmed the absence of library chimeras.

Golden Gate assembly (Engler & Marillonnet, 2013) was used to combine the variable RNA barcode and sgRNA spacer sequences (flanked by GG priming sequences) with the plasmid backbone. For specific amplification of library subpools, the Golden Gate adaptors are flanked by 20‐nt primer binding sequences (see upper section of Fig EV1). The RNA barcode is expressed from the strong T7 promoter and transcriptionally fused to the 5′ end of the stable structural d0 RNA (Delebecque *et al*, 2011). The first two guanines of the consensus T7 transcript were kept fixed to ensure strong expression (Imburgio *et al*, 2000). The expression of the sgRNA is driven by the synthetic constitutive promoter J23101 (iGEM Registry of Standard Biological Parts). The putative transcriptional start site of J23101 was kept fixed with an adenine, which was found to be favored (Vvedenskaya *et al*, 2015). The pGuide plasmid backbone, which provides kanamycin resistance (kanR) and contains a high copy number pUC origin of replication, was designed to contain the minimal sequences required for selecting and replicating the dual RNA expression cassette (Fig EV2).

### Bulk growth rate and CRISPRi repression assay

To investigate the bulk growth and CRISPRi characteristics of the DuMPLING proof‐of‐principle system, the P1‐lacY, P2‐lacI, and P3‐control pGuide plasmids in the BW25993 *intC::tetR‐dcas9‐aadA lacY::ypet‐cat araB::T7 RNAP‐tetA* Δ*araB* screening strain were assayed for growth (OD_600_) and YPet fluorescence using an Infinite M200 plate reader (Tecan).

#### Cultures

Overnight cultures of the wild‐type BW25993 strain with the empty pGuide plasmid, the DuMPLING screening strain with the empty pGuide plasmid, the P1‐lacY, P2‐lacI, and P3‐control pGuide plasmids were grown in LB + 50 μg/ml kanamycin at 37°C shaking at 200 rpm.

#### Pre‐plate

In the morning, overnight cultures were diluted 1:400 into 200 μl supplemented M9 medium [100 μM CaCl_2_, 2 mM MgSO_4_, 1× M9 salts, 0.8% v/v glycerol, 1× RPMI amino acid mix (Sigma)] + 50 μg/ml kanamycin + 0.85 g/l Pluronic F108 in a transparent 96‐well plate with lid (Costar Assay Plate, REF 3370, Corning). LacY‐Ypet and dCas9 were induced by adding isopropyl β‐D‐1‐thiogalactopyranoside (IPTG) (1 mM final concentration) and anhydrotetracycline (aTc) (1 ng/μl final concentration), respectively. To control for the EtOH in the aTc stock, 100 ppm EtOH was added to media without aTc. Plate reader cultures were grown at 37°C, with shaking (1 min, 4.5 mm amplitude) and measurements (OD_600_ and fluorescence with 510 ± 9 nm excitation and 540 ± 20 nm emission) every 5 min.

#### Experiment run

The pre‐plate cultures were diluted 1:200 once they hit exponential phase and run for 20 h as described above (Costar Assay Plate, REF 3904, Corning).

#### Analysis

The raw data were analyzed using custom MATLAB scripts. The maximum growth rates were converted to minimum doubling times (Fig EV3A). After subtracting the medium background absorption and fluorescence, the fluorescence was normalized with OD_600_ (Fig EV3B) and these values were used for calculating CRISPRi repression ratios.

#### Results

*P1‐lacY sgRNA:* LacY‐YPet was repressed 19.8‐fold upon dCas9 induction. The leakage repression was negligible compared with the empty vector control. *P2‐lacI sgRNA:* LacY‐YPet expression was activated to 24.1‐fold over the cell background level and 0.43‐fold of the maximal IPTG induction levels in the empty vector control, due to suppression of LacI expression. *P3‐control strain:* The ratio of LacY‐YPet expression with the empty vector culture was close to 1 (1.04 for induction with both IPTG and aTc, 0.90 for just aTc).

These data illustrate the low leakage of dCas9 expression in the DuMPLING screening strain, which is important to avoid biasing the screening population before phenotyping.

### The microfluidic chip

The microfluidic chip is a PDMS (Polydimethylsiloxane)–glass hybrid disposable device where the flow is driven by pressure. We describe the microfluidic chip design, production, and operation in Baltekin *et al* (2017). The chip is designed to rapidly capture individual bacterial cells from liquid growth cultures and exchange the liquid media around the cells effectively while keeping the captured cells in place throughout the experiment. Here, the chip design enables effective delivery and exchange of different media, probes, and buffers during the genotyping.

### Microscope setup

All imaging were carried out using a Nikon Ti‐E setup for both phase contrast and epifluorescence microscopy. The microscope was equipped with 100× CPI Plan Apo Lambda (Nikon). Phase contrast images were acquired using a dmk23u274 (The Imaging Source). Bright‐field and fluorescence images were acquired using a Zyla 4.2 PLUS sCMOS (Andor).

For wide‐field epifluorescence‐based phenotyping, a 300 ms excitation [shuttered using an AOTFnC (AA Opto Electronics)] from a 514‐nm CW‐laser at 415 W/cm^2^ (Fandango, Cobolt) was used. The laser light was reflected on a zt514.5rdc (Chroma) dichroic before hitting the sample. The Ypet emitted light was transmitted through the above dichroic and filtered through a BrightLine Fluorescence 542/27 (Semrock) before hitting the sCMOS camera. The genotyping and DAPI imaging were carried out using LED white light source (Sola, Lumencore) together with the appropriate filter cubes. Filter cube for Cy3 detection: excitation filter: FF01‐543/22 (Semrock), dichroic mirror: FF562‐Di03 (Semrock), emission filter: FF01‐586/20 (Semrock). Filter cube for Cy5 detection: excitation filter: FF01‐635/18 (Semrock), dichroic mirror: FF652‐Di01 (Semrock), emission filter: FF01‐680/42 (Semrock).

### Loading cells into the microfluidic chip

Overnight cultures of the strains to be loaded were grown in LB + 50 μg/ml kanamycin at 37°C shaking at 200 rpm. In the morning, cells were diluted 1:200 in M9 + 0.2% Glucose + 1× RPMI + 50 μg/ml kanamycin + 0.85 g/l Pluronic F108 and grown for 2 h at 37°C shaking at 200 rpm, at which point cells were flown into the chip and into the cell channels where they are caught by the 300 nm constriction at the end of the cell channels [as described in (Baltekin *et al*, 2017)]. The cells were grown in the chip overnight in M9 + 0.2% Glucose + 1× RPMI + 50 μg/ml kanamycin + 0.85 g/l Pluronic + 0.1 ng/μl aTc at 30°C, and then imaged.

### Imaging phenotypes

Cells were imaged for 272 min in the same conditions as overnight growth. Phase contrast images were taken every minute. Bright‐field images and epifluorescence images were taken every 13 min. Microscope and accessory equipment were controlled using micro‐manager (version 1.4.20) (Edelstein *et al*, 2010).

### Genotyping by sequential FISH

After phenotype imaging was complete, the media was switched to LB + 20% arabinose + 50 μg/ml kanamycin + 0.85 g/l Pluronic, and the cells were grown 3 h further at 30°C. After arabinose induction, the cells were fixed in a solution of 1× PBS + 4% formaldehyde for 10 min at room temperature (all steps from this point forward were carried out at room temperature). The cells were then washed with 1× PBS + Ribolock (Thermo Scientific). The cells were then permeabilized with 70% EtOH for 45 min. The 70% EtOH was washed away with 50% EtOH, then 25% EtOH, and finally with 1× PBS + Ribolock.

For each round of FISH, the appropriate probe pool was flowed into the chip [30 μl hybridization probes + 7.5 μl Ribolock (Thermo Scientific) + 30 μl *E. coli* tRNA (0.65 mg/ml) + 233 μl (0.05 g/ml Dextran sulfate sodium salt, 20% formamide and 2× SSC)]. Hybridization was allowed to proceed overnight (~16 h). The excess probes were washed away with PBS + DAPI stain + Ribolock and then imaged in DAPI, Cy3, and Cy5 using the white light source (SOLA). After imaging, the cells were incubated in a solution of 90% formamide + 2× SSC for 1 h to wash away bound probes and then washed again in PBS + DAPI stain + Ribolock to remove the previous reagents. The cells were again imaged as before to ensure that the probes were fully removed. This was the completion of one round of probing, and at this point, the next pool of probes was flowed into the chip.

### Fluorescent *in situ* hybridization probe production

**Figure EV5:**
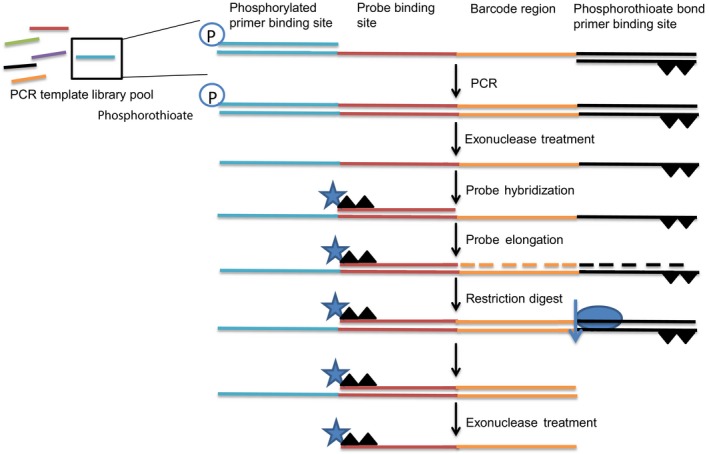
Schematic overview of the enzymatic steps of the probe generation protocol Templates were amplified from the template library pool by PCR using primers specific for each FISH genotyping round. Lambda exonuclease selectively digested the 5′‐phosphorylated strand, leaving only the 5′‐phosphorothioate strand. Fluorescently labeled and phosphorothioate‐modified elongation probes were hybridized to the ssDNA template and elongated with DreamTaq polymerase. The dsDNA product was digested with restriction enzyme SchI, removing the phosphorothioate bonds from the unlabeled strand. Lambda exonuclease digestion produced the final FISH probe.

The steps for probe production, adapted from Beliveau *et al* and Chen *et al* (Beliveau *et al*, 2012; Chen *et al*, 2015), are seen in Fig EV5. Sequences can be found in Table EV2. Templates for FISH probe elongation rounds 1 and 2 (P1 R1 E0, P2 R1 E1, P3 R1 E0 or P1 R2 E1, P2 R2 E0, P3 R2 E0) were pooled separately and PCR‐amplified with DreamTaq polymerase using phosphorylated forward primers and phosphorothioate‐modified reverse primers (R1 FWD and R1 REV or R2 FWD and R2 REV). The PCR product was purified using the PureLink quick PCR purification kit (Invitrogen). The phosphorylated strand was selectively digested by lambda exonuclease (Thermo Scientific) treatment for 30 min at 37°C followed by heat inactivation at 80°C for 10 min. The ssDNA was purified using the MinElute PCR purification kit (Qiagen). The ssDNA template was elongated by hybridization of the corresponding phosphorothioate‐modified Cy3 or Cy5 elongation probes (E0 Cy3 and E1 Cy5) at 55°C for 5 min after an initial heating step at 96°C for 3 min. Elongation was performed with DreamTaq polymerase and dNTP in DreamTaq buffer at 72°C for 15 min. The elongated product was purified using the PureLink quick PCR purification kit (Invitrogen) and cleaved by the SchI FD enzyme for 30 min at 37°C. After this step, lambda exonuclease was added directly to the SchI digestion for an additional 30 min at 37°C. The processed FISH probes were purified using phenol/chloroform/isoamylalcohol (VWR), washed with chloroform (Sigma‐Aldrich), and extracted by means of centrifugation after precipitation with EtOH and sodium acetate. The DNA pellet was washed once in 70% EtOH and dried at room temperature before being resolved in water. To remove any additional undesirable DNA, the probe mixture was purified on a 4% agarose gel. The expected DNA band was excised from the gel, sliced in small pieces, and incubated overnight in water. The extracted probe was phenol/chloroform/isoamylalcohol‐purified, washed, and extracted as previously described followed by filtration in Ultrafree‐MC microcentrifuge filters (Sigma‐Aldrich) before being used in the microfluidic experiment.

### Polyacrylamide gel analysis of produced probes

**Figure EV6:**
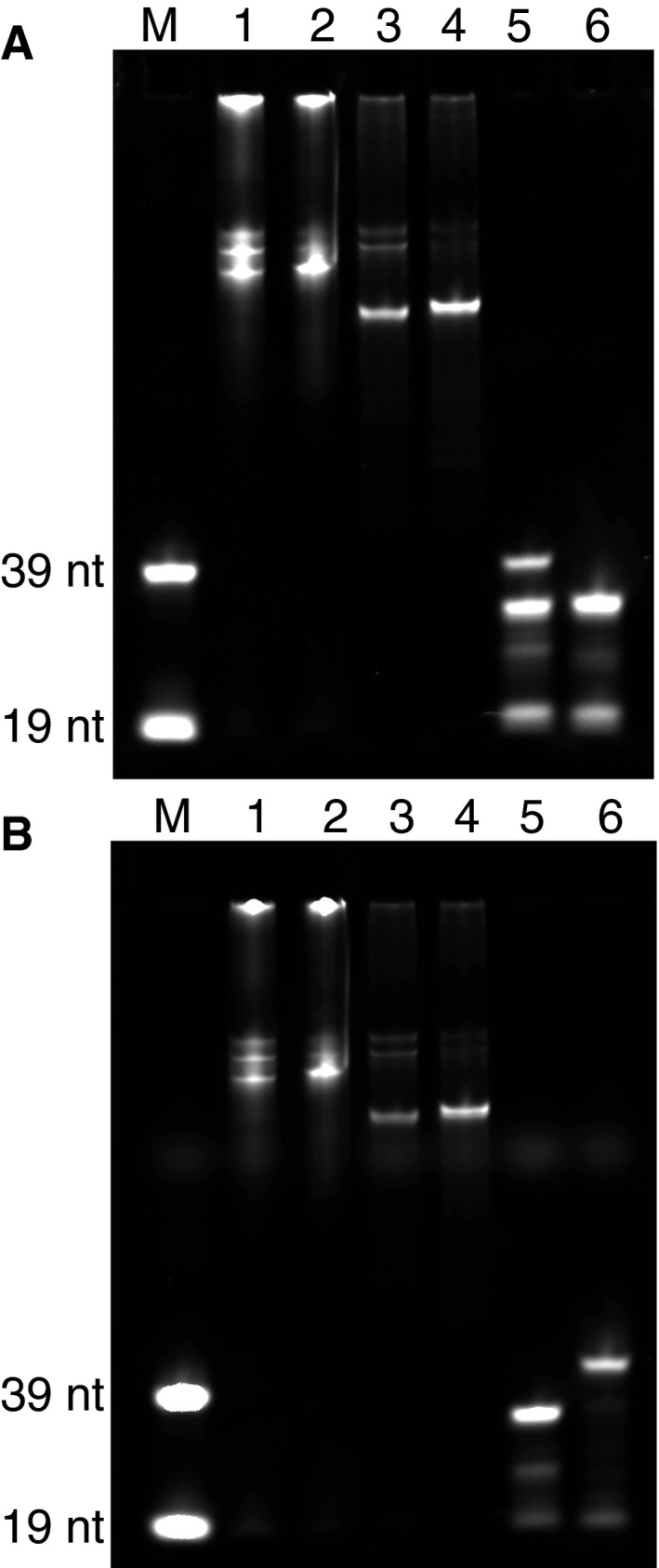
Products from the different steps of the FISH probe production protocol A, BProducts were run on a 10% polyacrylamide gel and imaged in (A) Cy3 and (B) Cy5 channels. (M): Cy3‐ and Cy5‐labeled 39‐nt and 19‐nt ssDNA probes were used as size references. (1) and (2): the initial fluorescent elongation product for the two rounds of FISH probe generation, respectively. (3) and (4): SchI digestion of the elongation products for rounds one and two, respectively. (5) and (6): Lambda exonuclease treatment and gel‐purified product for rounds one and two, respectively.

Samples were collected throughout the probe production protocol, mixed with 10× FD green buffer, and loaded on a 10% polyacrylamide gel (Bio‐Rad). As size references, Cy3 and Cy5 39‐nt ssDNA probes with two phosphodiester bonds, and also the Cy3 and Cy5 19‐nt probes used for elongation, were loaded onto the gel. The gel was run in 1× TBE buffer in a Mini‐PROTEAN system (Bio‐Rad) and analyzed with a Chemidoc system (Bio‐Rad) (Fig EV6).

### Image analysis

#### Phenotyping

Cell outlines were identified using cell segmentation (Ranefall *et al*, 2016) of phase contrast images. Before segmentation, the image of a trap designed to be without cells was deducted from all traps imaged in phase contrast as described in Baltekin *et al* (2017). Using the detected cell outlines, lineages were constructed using the Baxter algorithm (Magnusson *et al*, 2015) where Jaccard indices between consecutive cell outlines were used to score migration and division events. The division event scores were calculated to require binary fission. Cell lineages from Baxter were filtered based on the following criteria: (i) Cell outlines where the size transiently dropped or increased by large amounts were deemed as missegmentation and not used in further analysis. (ii) Lineages from one cell generation with large shifts in size (non‐transient) were excluded from further analysis. (iii) Lineages from one cell generation with large center of mass movements of the cell outlines were excluded from further analysis. Finally (iv), lineages from one cell generation with very short life span were excluded from further analysis unless they contained both a mother and two daughter cells. Fluorescently labeled LacY‐YPet molecules were localized using the dot detection algorithm suggested by Loy and Zelinsky (Loy & Zelinsky, 2003). Given that phase contrast and fluorescence images were acquired using different cameras, a transformation was required to place dots inside segmented cell outlines. This transformation was estimated before the start of the experiment using landmarks in images captured on the two different cameras.

#### Genotyping

DAPI, Cy3, and Cy5 images were summed vertically (see Fig 3B). The locations of the cell traps were determined using the vertically summed DAPI signal. The log ratio of vertically summed Cy3 and Cy5 signals was used to call a 1 or 0. The genotype of the trap was then associated with all cells in that trap.

### Sensitivity and specificity

The bright fluorescent phenotype is easy to identify, which makes it possible to use this as a reference when calculating sensitivity and specificity for the genotyping in our experiment. There were 74 traps with bright cells and 159 with the other strains. One of the 1,398 attempts to read a barcode gave the wrong answer, which would lead to a misclassification of a bright trap as a non‐bright trap. However, this could be corrected since only two rounds of probing are needed to call the genotype and we probed six rounds. This implies that each genotype has been determined 6/2 = 3 times.

In terms of sensitivity and specificity, the true‐positive identifications of the bright genotype was made (74 × 3) − 1 = 224 times. The true‐negative identifications of the bright genotype was made 159 × 3 = 477 times. There is one false negative and 0 false positive. Based on this, we calculated the 95% Clopper–Pearson confidence intervals for the sensitivity to be 97.55–99.99% and for specificity to be 99.23–100.00% (Clopper & Pearson, 1934).

If this experiment is used as a proxy for a library that requires six rounds of probing to identify each genotype, then the true‐positive identification of the bright genotype was made 74 − 1 = 73 times. The true‐negative identification of the bright genotype was made 159 times. There is one false negative and 0 false positives. Based on this, we calculated the 95% confidence intervals for the sensitivity to be 92.7–99.97% and for specificity to be 97.71% to 100%.

### Note on chemicals and reagents

All chemicals were acquired from Sigma‐Aldrich unless otherwise stated. All synthetic DNAs are from Integrated DNA Technologies, and unlabeled DNA oligonucleotides above 100 nt were bought as Ultramers. DreamTaq DNA polymerase (Thermo Scientific) was used for colony PCRs, PCRs for sequencing reactions, preparative PCRs of small fragments (< 200 bp), and emulsion PCR. Phusion DNA polymerase (Thermo Scientific) was used for preparative PCRs. For difficult preparative PCRs, PrimeSTAR DNA polymerase (Takara) was used. For preparative PCRs requiring extra high accuracy, Q5 RNA polymerase was used (NEB). Restriction enzymes, ligases, and other cloning‐related enzymes were procured from Thermo Scientific unless otherwise stated.

### Data availability

The code used for analyzing the data and generating images is provided as Code EV1. Raw images can be downloaded from BioStudies https://www.ebi.ac.uk/biostudies/ (accession code: S‐BSST37).

## Author contributions

JE conceived the concept and coordinated the project. MJL developed the phenotyping and genotyping protocols and carried out the corresponding experiments. DC designed the strains and made them. JL and MJL developed the probe synthesis method. ÖB developed the microfluidic device. DF and MJL developed the microscopy and analysis methods. JE, MJL, DF, DC, and JL wrote the manuscript. However, the authors worked closely on the whole project and made substantial contributions in each other's main areas.

## Conflict of interest

Concepts related to this work are described in the patent application PCT/SE2015/050227.

## Supporting information



Expanded View Figures PDF

Table EV1

Table EV2

Movie EV1

Movie EV2

Code EV1

Review Process File
