# Supplementary material for: In situ genotyping of a pooled strain library after characterizing complex phenotypes
Source: Mol Syst Biol. 2017 Oct 17;13(10):947. doi: 10.15252/msb.20177951 (PMC5658705; doi:10.15252/msb.20177951)
Supplement: Supplementary file 1 — Expanded View Figures PDF [file MSB-13-947-s001.pdf]

Expanded View Figures

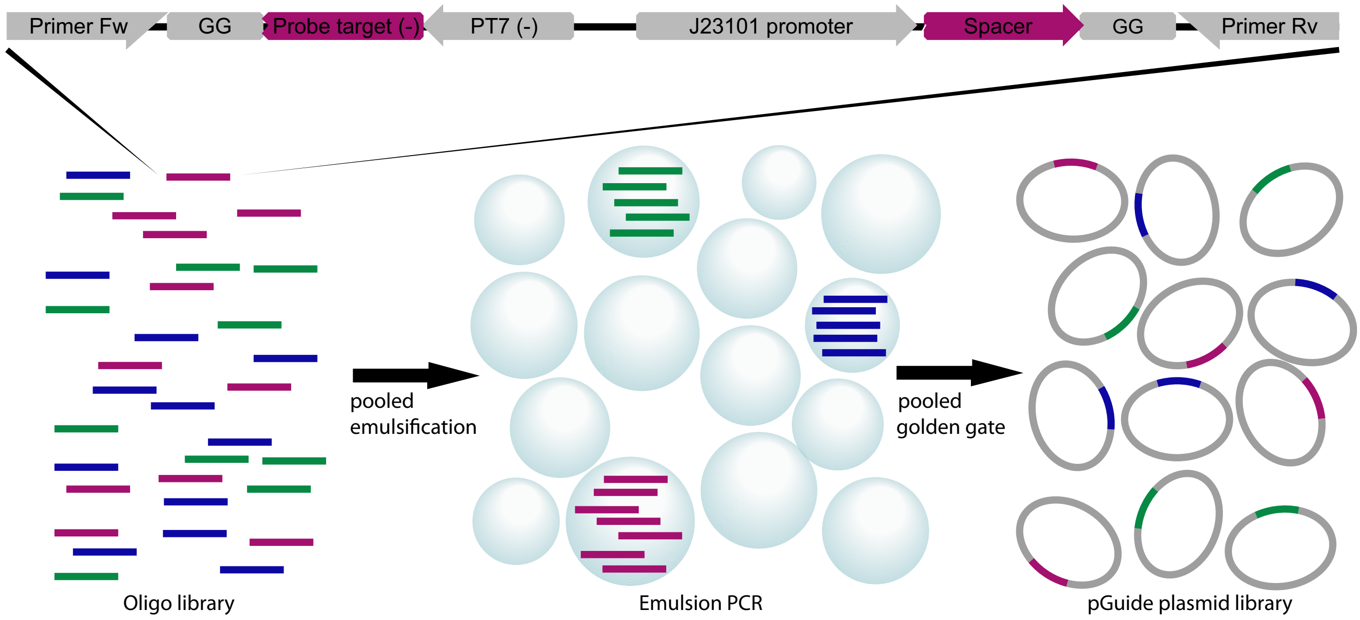

Figure EV1. DuMPLING oligonucleotide plasmid library design and production.

Each member of the oligonucleotide library for plasmid construction contains two divergent promoters: a constitutive promoter toward the spacer and PT7 toward the barcode RNA (top). The oligonucleotide library was amplified using pooled emulsion PCR to avoid the formation of chimeras (bottom middle). The pool of amplified oligonucleotides was assembled into a functional pGuide plasmid library using pooled Golden Gate (bottom right).

 © 2017 The Authors

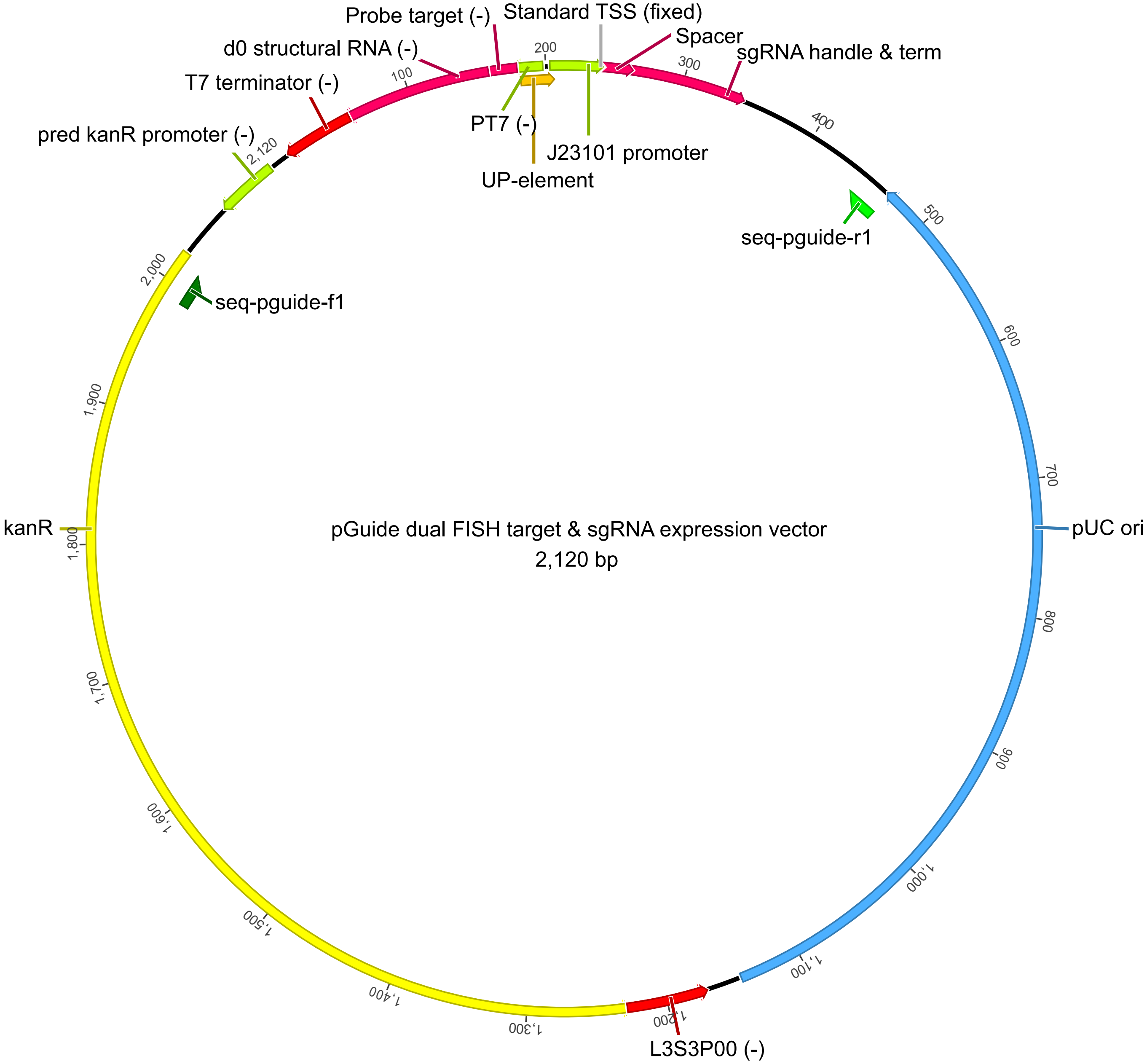

Figure EV2. Sequence elements of the dual RNA barcode FISH target and sgRNA expression pGuide vector.
Minus (−) signs indicate anti-sense direction of genetic element.

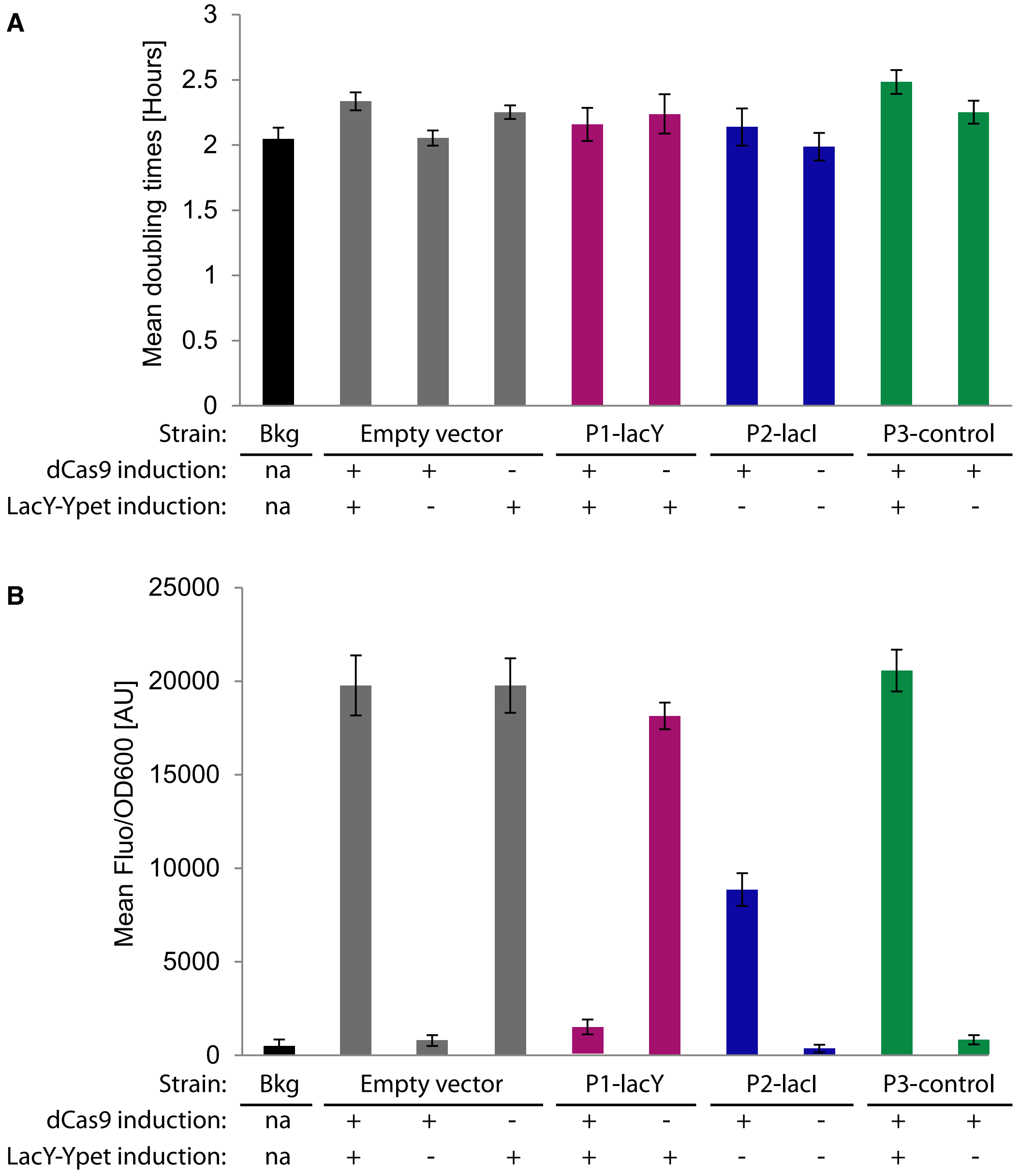

Figure EV3. Bulk growth rate and CRISPRi repression assay results of the DuMPLING screening strain with different pGuide constructs.

A Mean doubling times of the different strains under the indicated conditions.

B Steady-state mean fluorescence normalized by cell density (Fluo/OD_{600}) of the different strains under the indicated conditions.

Data information: Error bars indicate sample standard deviations (Bkg *n* = 3, Empty vector *n* = 3, P1-lacY and P2-lacI *n* = 6, P3-control *n* = 5). Bkg (cell background), AU (arbitrary units), na (not applicable).

 © 2017 The Authors

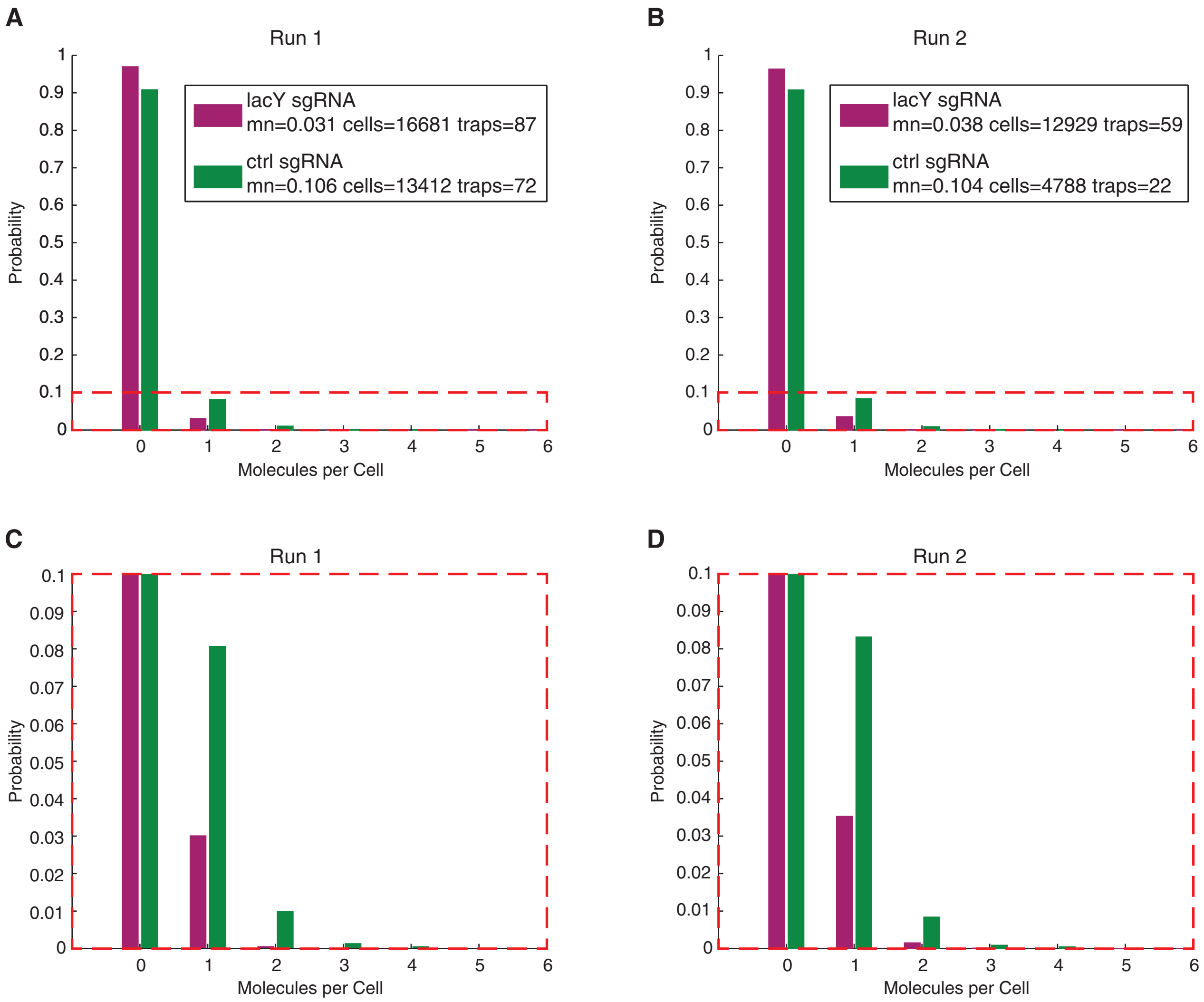

Figure EV4. Reproducibility of dot detection results.

A–D Normalized histograms of single-molecule counting of expression from the two low-expression genotypes (strain definitions are given in the Materials and Methods section "Design and construction of the CRISPRi/RNA barcode plasmid library"). (A and C) are the same results displayed in Fig 4B. (B and D) are from a repeat of the same experiment 1 week later. Note: (A and B) are the full histogram, (C and D) are zoom in on the lower frequency events. mn: mean.

© 2017 The Authors

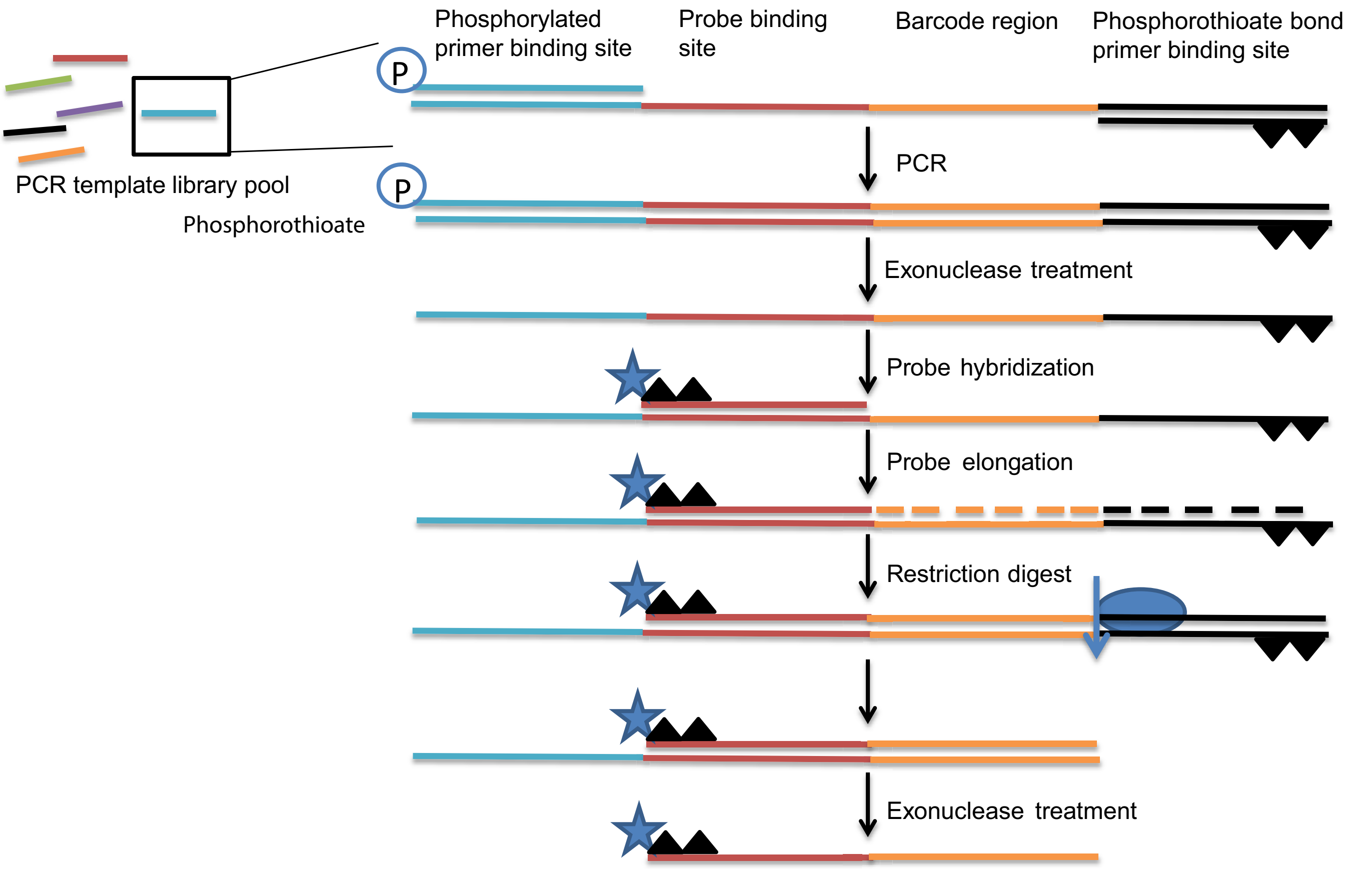

Figure EV5. Schematic overview of the enzymatic steps of the probe generation protocol.

Templates were amplified from the template library pool by PCR using primers specific for each FISH genotyping round. Lambda exonuclease selectively digested the 5′-phosphorylated strand, leaving only the 5′-phosphorothioate strand. Fluorescently labeled and phosphorothioate-modified elongation probes were hybridized to the ssDNA template and elongated with DreamTaq polymerase. The dsDNA product was digested with restriction enzyme SchI, removing the phosphorothioate bonds from the unlabeled strand. Lambda exonuclease digestion produced the final FISH probe.

 © 2017 The Authors

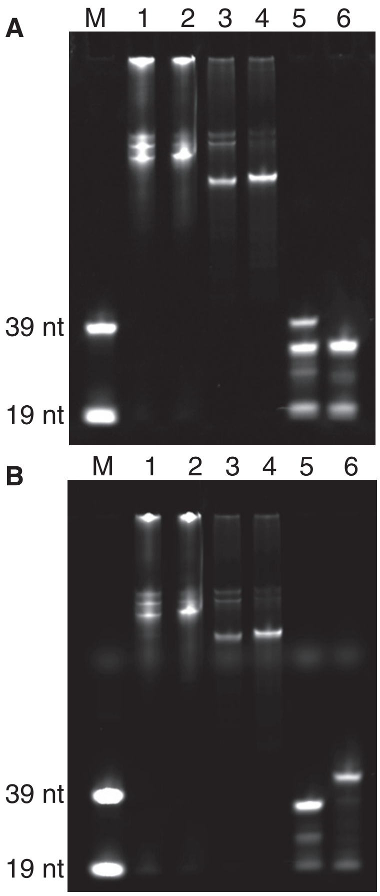

Figure EV6. Products from the different steps of the FISH probe production protocol.

A, B Products were run on a 10% polyacrylamide gel and imaged in (A) Cy3 and (B) Cy5 channels. (M): Cy3- and Cy5-labeled 39-nt and 19-nt ssDNA probes were used as size references. (1) and (2): the initial fluorescent elongation product for the two rounds of FISH probe generation, respectively. (3) and (4): SchI digestion of the elongation products for rounds one and two, respectively. (5) and (6): Lambda exonuclease treatment and gel-purified product for rounds one and two, respectively.

© 2017 The Authors
