# Supplementary material for: In situ genotyping of a pooled strain library after characterizing complex phenotypes
Source: Mol Syst Biol. 2017 Oct 17;13(10):947. doi: 10.15252/msb.20177951 (PMC5658705; doi:10.15252/msb.20177951)
Supplement: Supplementary file 5 — Movie EV2 [file MSB-13-947-s005.zip › MSB_7951_Legend_Movie_EV2.docx]

**Legend Movie EV2**

**Fluorescence movie during phenotyping.**
